# Fecal Microbiota Transplantation Modulates the Gut Flora Favoring Patients With Functional Constipation

**DOI:** 10.3389/fmicb.2021.700718

**Published:** 2021-10-07

**Authors:** Xueying Zhang, Ning Li, Qiyi Chen, Huanlong Qin

**Affiliations:** Intestinal Microenvironment Treatment Center, Shanghai Tenth People’s Hospital, Tongji University School of Medicine, Shanghai, China

**Keywords:** fecal microbiota transplantation, gut microbiome, functional constipation, 16S rDNA gene sequencing, short chain fatty acid, serum inflammatory factor

## Abstract

Intestinal dysmotility is common in many diseases and is correlated with gut microbiota dysbiosis and systemic inflammation. Functional constipation (FC) is the most typical manifestation of intestinal hypomotility and reduces patients’ quality of life. Some studies have reported that fecal micriobiota transplantation (FMT) may be an effective and safe therapy for FC as it corrects intestinal dysbiosis. This study was conducted to evaluate how FMT remodels the gut microbiome and to determine a possible correlation between certain microbes and clinical symptoms in constipated individuals. Data were retrospectively collected on 18 patients who underwent FMT between January 1, 2019 and June 30, 2020. The fecal bacterial genome was detected by sequencing the V3–V4 hypervariable regions of the 16S rDNA gene. Fecal short chain fatty acids (SCFAs) were detected by gas chromatography-mass spectrometry, and serum inflammatory factor concentrations were detected via enzyme-linked immunosorbent assay. Comparing the changes in fecal microbiome compositions before and after FMT revealed a significant augmentation in the alpha diversity and increased abundances of some flora such as Clostridiales, *Fusicatenibacter*, and *Paraprevotella*. This was consistent with the patients experiencing relief from their clinical symptoms. Abundances of other flora, including *Lachnoanaerobaculum*, were decreased, which might correlate with the severity of patients’ constipation. Although no differences were found in SCFA production, the butyric acid concentration was correlated with both bacterial alterations and clinical symptoms. Serum IL-8 levels were significantly lower after FMT than at baseline, but IL-4, IL-6, IL-10, and IL-12p70 levels were not noticeably changed. This study showed how FMT regulates the intestinal microenvironment and affects systemic inflammation in constipated patients, providing direction for further research on the mechanisms of FMT. It also revealed potential microbial targets for precise intervention, which may bring new breakthroughs in treating constipation.

## Introduction

Intestinal motility disorder is common with many diseases such as irritable bowel syndrome (IBS), inflammatory bowel diseases, critical illness, and postsurgical intestinal dysfunction. Intestinal dysmotility alone may lead to poor quality of life for patients, while dysmotility in other diseases may contribute to a worse prognosis (Garrett and Bar-Or, 2008, Shimizu et al., 2011). Thus, gut motility disorder is problematic and requires further study.

Intestinal dysmotility is associated with many factors, including neuroimmune interactions and gut microbiota changes (De Jonge et al., 2005; Zhao and Yu, 2016). The intestinal flora may regulate intestinal motility by releasing bacterial metabolites such as short-chain fatty acids (SCFAs), intestinal neuroendocrine factors, and mediators released by the gut immune response (Barbara et al., 2005). Long durations of intestinal dysmotility, such as with chronic constipation, may alter the microbiome composition and intestinal permeability, which may lead to systemic immune system activation and inflammatory status changes (Khalif et al., 2005; de Jong et al., 2016). Therefore, intestinal motility disorders involve a series of complex pathophysiological processes in which the gut microbiota may play a key role.

Functional constipation (FC) is the most typical manifestation of intestinal hypomotility. According to the ROME IV criteria, FC is a gut-brain interaction disorder (Drossman and Hasler, 2016). Patients have difficulty defecating and may experience depression and/or anxiety. Furthermore, severe constipation can potentially lead to bowel obstruction. The American Gastroenterological Association recommends microbiota-modulating methods, such as dietary control and fiber intake, as the first-line treatment for FC, and probiotics such as *Bifidobacterium lactis* DN-173 010 and *Lactobacillus casei* Shirota are reported to have potential treatment efficacy (Chmielewska and Szajewska, 2010; Mearin et al., 2016). Laxatives and 5-HT4 receptor agonists are also recommended as empiric therapy (Mearin et al., 2016). However, few conservative treatments exist for refractory constipation, and patients whose symptoms are not relieved by the above treatments may require surgery.

Fecal microbiota transplantation (FMT) may help cure constipation that cannot be alleviated by other conservative treatments and may help many patients (Tian et al., 2017; Tian Y. et al., 2020). FMT involves transferring the gut microbiotas from healthy donors to patients to treat diseases and is effective for recurrent or refractory *Clostridium difficile* infections (Cammarota et al., 2017; Mullish et al., 2018). Under approval from the Ethics Committee of Shanghai Tenth People’s Hospital of Tongji University, we have been performing FMT on patients with bowel disorders since 2017. As of June 2020, we have treated over 1,000 patients with constipation, with an efficacy rate of > 67%. Most constipated patients have other diseases, such as diabetes, Parkinson’s disease and mental disorders, or have histories of drug use or surgeries that contributed or might have contributed to their defecation difficulties. The underlying mechanism by which FMT regulates gut motility remains unclear, especially when combined diseases may also affect the pathophysiological processes of constipation. We conducted this retrospective study to evaluate the clinical efficacy and gut microbiota remodeling ability of FMT on constipated patients and to explore the potential mechanisms underlying FMT and gut motility. We attempted to build an appropriate model of bowel hypomotility by selecting FC patients without combined diseases or surgical histories or histories of using drugs that might influence bowel motility or the gut microbiome. We hypothesized that FMT could relieve constipation symptoms by remodeling the gut microbiota composition and that the possible mechanism might be correlated with altered abundances of key bacteria and altered metabolism of products such as SCFAs. FMT may affect patients’ systemic immunity, which might cause and/or result in motility changes.

## Materials and Methods

### Patient Clinical Data and Sample Collection

We retrospectively evaluated 18 patients with refractory constipation who were strictly without other combined diseases and were treated with FMT at Tenth People’s Hospital of Tongji University between January 1, 2019 and June 30, 2020. The Ethics Committee of Shanghai Tenth People’s Hospital of Tongji University reviewed and approved the study. Patients provided written informed consent to participate.

Patients were eligible for inclusion if they were diagnosed with FC according to the Rome IV criteria (Drossman and Hasler, 2016; Mearin et al., 2016) and had a Bristol Stool Form Scale (BSFS) of 1 or 2 (Lewis and Heaton, 1997). Other inclusion criteria were that patients were aged 18–65 years and had a body mass index (BMI) of 18–25 kg/m^2^.

Patients were excluded if they were pregnant or breast-feeding; their constipation was secondary to other diseases (e.g., endocrine, metabolic, or neurological disorders) or intervention (e.g., drugs); they had histories of organic digestive system diseases or disorders (e.g., peptic ulcers, bleeding erosive gastritis, megacolon, cancer, inflammatory bowel disease, intestinal obstruction, or other); they had a history of organ surgery; they had a history of systemic diseases (e.g., endocrine, renal, cardiovascular, respiratory, or other); they were definitively diagnosed with a psychiatric disorder; they had an active infection; they were treated with probiotics, prebiotics, antibiotics, or proton pump inhibitors within the last 3 months; or they had a previous history of FMT within the last year. Patients with depression or anxiety symptoms were also excluded, defined by a Hamilton Depression Scale (HAMD) or Hamilton Anxiety Scale (HAMA) ≥ 7. Considering the gut-brain interaction, mood disorders may influence both the gut microbiome and intestinal neurons; thus, mood disorders were considered confounding factors in studying how FMT affects gut motility (Mayer et al., 2015).

### Study Design

This was a retrospective single-arm study to evaluate the changes in the gut microbiota and related biomarkers in FC patients who underwent FMT as well as the correlation between specific bacteria and clinical symptoms. Constipation-related clinical symptoms were evaluated both before and 4 weeks after FMT by complete weekly spontaneous bowel movements (CSBMs), stool consistency (BSFS) (Mearin et al., 2016), Wexner constipation score, the Patient Assessment of Constipation-Symptoms (PAC-SYM), and the Patient Assessment of Constipation Quality of Life (PAC-QOL) questionnaire (Ding et al., 2018; Zhang et al., 2018). Patients were defined as clinically cured if they had an average of three or more CSBMs per week during follow-up (Tian et al., 2017). Stool and blood specimens were collected during clinical evaluation. Feces were stored at −80°C within 20 min after collection. Serum samples were collected between 6 and 8 a.m. and stored at −80°C before use. The efficacy of FMT for treating constipation was evaluated by comparing clinical symptoms before and after FMT. The influence of FMT on gut microbiota profiles and systemic inflammatory conditions was reflected by changes in the fecal flora composition, SCFA concentrations, and serum IL-4, IL-6, IL-8, IL-10, and IL-12p70 levels.

### Fecal Micriobiota Transplantation Process

#### Donor Screening

Unrelated donors were selected under the following conditions: (1) aged 18–30 years; (2) BMI of 18–25 kg/m^2^; (3) no pathological signs during physical examination; (4) no history or recent history of infectious diseases or gastrointestinal, metabolic, neurological, or other systematic disorders (Cammarota et al., 2017); (5) no recent use of drugs that can impair the gut microbiota composition (Cammarota et al., 2017); (6) had a regular routine and a healthy diet, appropriate exercise, family harmony, and no smoking or drinking habits; (7) passed blood and stool tests 4 weeks before donation, including general blood and stool testing and tests for possible pathogens or infectious diseases (Cammarota et al., 2017). Four donors met these criteria.

#### Preparation of Fecal Material

Approximately 100 g of donor feces were collected in a sterile container, mixed with 300 mL of sterile, non-bacteriostatic normal saline, passed through 2.0- and 0.5-mm sieves, amended with sterile glycerol to a final concentration of 10%, and stored frozen at −20°C for 1–8 weeks until use (Hamilton et al., 2012). Samples were prepared anaerobically within 6 h after fecal collection by the donor. The final fecal suspension contained the entire spectrum of the donor fecal microbiome as well as the metabolites and other possible active substances in the donor stool.

#### Clinical Management and Fecal Delivery

Patients orally received vancomycin for 3 days and underwent bowel lavage with polyethylene glycol 12–24 h before FMT. Fecal suspensions were thawed in a 37°C water bath and infused within 6 h of thawing via an indwelling nasojejunal tube (Cammarota et al., 2017). Each recipient received approximately 33.3 g of donor feces once daily from the same donor for 6 consecutive days (Zhang et al., 2018).

### Sample Testing

#### Fecal Microbiome Testing Based on 16S rDNA Gene Analysis

Patients were asked to empty their bladder, then deliver stool into a sterile container. Approximately 2 g of feces were taken from the central part of the stool that had no contact with the air and placed in sterile cryotubes for storage at −80°C.

The fecal DNA was extracted and quantified using a NanoDrop 2000 spectrophotometer (Thermo Fisher Scientific Inc., MA). The V3-V4 hypervariable regions were amplified via polymer chain reaction (PCR) using the universal primers, forward (5′–3′): CCTACGGGRSGCAGCAG (341F) and reverse (5′–3′): GGACTACVVGGGTATCTAATC (806R). The amplicons were purified using an AxyPrep DNA Gel Extraction Kit (Axygen Biosciences, Union City, CA) followed by library quantification using a Qubit^TM^ dsDNA BR Assay Kit (Thermo Fisher Scientific). Finally, the pooled amplicons were paired end sequenced (2 × 250 bp) on an Illumina HiSeq PE250 sequencing platform. The sequencing depth was 42,501 reads per sample.

#### Fecal Short Chain Fatty Acid Quantification

Fecal SCFAs were quantified as previously described (Zheng et al., 2013). The fecal supernatant samples were quantified using an Agilent 7890A gas chromatograph coupled with an Agilent 5975C mass spectrometric detector (Agilent Technologies, U.S.). The initial oven temperature was 90°C, which was increased to 120°C at 10°C/min, to 150°C at 5°C/min, to 250°C at 25°C/min, and finally held at 250°C for 2 min. The concentrations of acetic acid (3.209 min), propionic acid (3.974 min), isobutyric acid (4.265 min), butyric acid (4.954 min), isovaleric acid (5.511 min), valeric acid (6.394 min), and caproic acid (8.023 min) were separated via a polar DB-WAX capillary column (30 m × 0.25 mm ID^∗^ 0.25 μm, Agilent, CA) with helium as the carrier gas at a constant flow rate of 1 mL/min.

#### Serum Inflammatory Factor Detection

Serum IL-4, IL-6, IL-8, IL-10, and IL-12p70 levels were detected using enzyme-linked immunosorbent assay (ELISA) kits (Neobioscience, China) according to the manufacturer’s protocol.

### Statistical Analysis

#### Analysis of 16S Sequencing Data

Raw FASTQ files were aligned using Pandaseq (version 2.7) and quality-filtered (Masella et al., 2012). The tags were clustered into operational taxonomic units (OTUs) with a 97% similarity cutoff using UPARSE (version7.1) (Edgar, 2013), and 868 OTUs were revealed. The taxonomy of each 16S rDNA gene sequence was analyzed using RDP Classifier^1^ and annotated to each classification level (kingdom, phylum, class, order, family, genus, and species). Microbiota richness was analyzed using QIIME. Alpha diversity was calculated and compared between the two groups (before and after FMT) using paired Wilcoxon tests. Beta diversity was evaluated by (un)weighted UniFrac distances and analyzed by ANOSIM. Significant differences in microbiota abundances were compared via linear discriminant analysis effect size (LEfSe).

#### Analysis of Other Data and Correlations With the Microbiota

Continuous data are presented as the mean ± standard deviation, hierarchical data are presented as the medium (minimum, maximum), and categorical data are presented as numbers (%). Paired *t*-tests and paired Wilcoxon tests were performed to determine the differences in clinical symptoms, SCFA concentrations, and inflammatory factors. Spearman correlations were used to identify relationships between the microbiota and other phenotypes. Analyses were performed using R (version 4.0.3). A two-tailed *p* < 0.05 was considered statistically significant.

## Results

### Patients’ Characteristics and Clinical Symptoms

Eighteen patients with constipation who were treated with FMT were included in the analysis. Table 1 and Supplementary Table 1 show the patients’ clinical characteristics. Patients’ constipation symptoms were significantly alleviated 4 weeks post-FMT (Table 2). Spontaneous defecation (CSBMs/week) increased from 0.7 ± 0.8 to 4.8 ± 2.5, and the stool form improved from hard to smooth and soft. Objective evaluation (Wexner score) and self-assessment (PAC-SYM, PAC-QOL) of the severity of constipation were significantly alleviated (*p* < 0.05). Supplementary Table 2 shows patients’ detailed clinical scale scores before and after treatment. Of the 18 patients, 14 were clinically cured, while the remaining 4 continue to experience constipation after the FMT; thus, the total success rate was 77.78%. Table 3 shows the donor-recipient pairs and clinical outcomes.

**TABLE 1:** Patient characteristics.

Characteristics	Eligible patients (*n* = 18)
Age (years)	45.1 ± 13.3
Gender (female)	13 (72.2%)
BMI (kg/m^2^)	22.0 ± 2.6
Disease duration (years)	10.3 ± 7.8
Combined diseases	None
HAMA	3.7 ± 1.8
HAMD	2.3 ± 2.0

**TABLE 2:** Evaluation of FC patients’ clinical symptoms before and after FMT.

Clinical symptoms	Group before (*n* = 18)	Group after (*n* = 18)	*P*-value
CSBMs/week	0.7 ± 0.8	4.8 ± 2.5	0.0007***
BSFS	1 (1.2)	4 (1.4)	0.0002***
Wexner score	12.7 ± 3.6	6.6 ± 5.6	<0.0001****
PAC-SYM	21.1 ± 6.5	7.9 ± 8.0	<0.0001****
PAC-QOL	50.2 ± 23.1	18.6 ± 18.1	0.0006***

*Group before: patients before FMT; Group after: patients 4 weeks after FMT.*

***0.0001 ≤ p < 0.001, ****p < 0.0001.

**TABLE 3:** Donor-recipient pairs and clinical outcomes.

ID	Donor	Clinical curation
Patient 1	Donor 1	No
Patient 2	Donor 1	No
Patient 3	Donor 1	Yes
Patient 4	Donor 1	No
Patient 5	Donor 2	No
Patient 6	Donor 2	Yes
Patient 7	Donor 2	Yes
Patient 8	Donor 2	Yes
Patient 9	Donor 2	Yes
Patient 10	Donor 2	Yes
Patient 11	Donor 2	Yes
Patient 12	Donor 3	Yes
Patient 13	Donor 3	Yes
Patient 14	Donor 3	Yes
Patient 15	Donor 3	Yes
Patient 16	Donor 4	Yes
Patient 17	Donor 4	Yes
Patient 18	Donor 4	Yes

### Microbial Community Diversity and Composition

**FIGURE 1:**
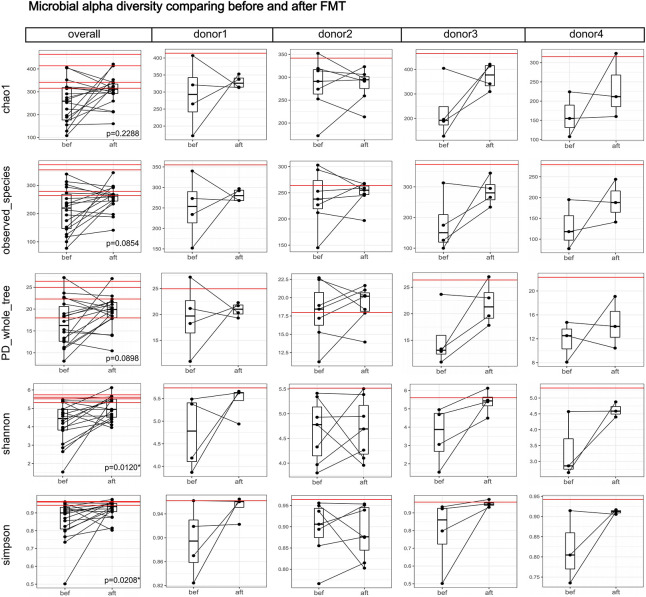
Boxplots comparing the gut microbial alpha diversities before and after FMT. Each black dot represents one sample (patient); pre-FMT and post-FMT samples from the same patient are connected by a black line. Each red horizontal line represents one donor. The “overall” column shows the alpha diversity indexes of the 18 patients and four donors, and the significance test between the before and after groups was a paired-samples Wilcoxon-test (“*” indicates 0.01 ≤ *p* < 0.05). The Shannon and Simpson indexes increased significantly after FMT. Columns for each donor show the alpha diversity changes in each donor-recipient pair. We did not perform separate significance tests on each donor-recipient pair because of the small sample size.

**FIGURE 2:**
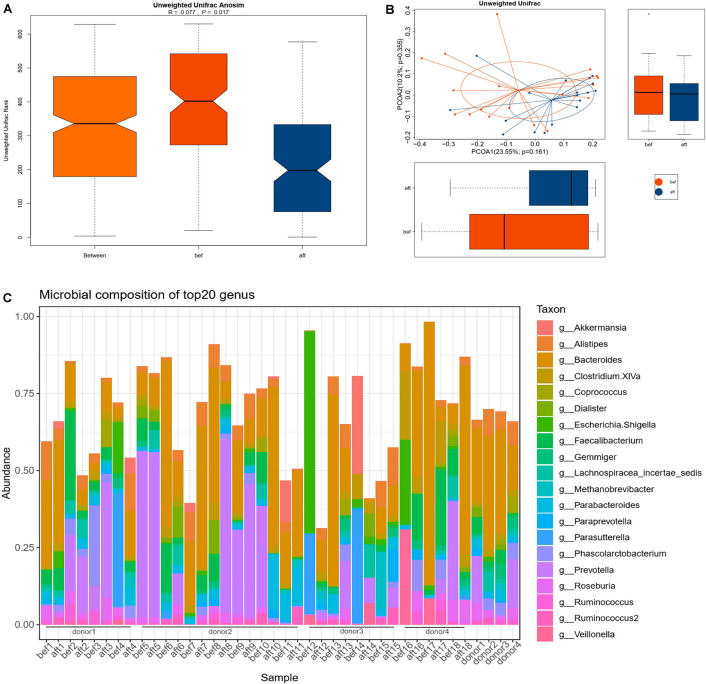
Bacterial community compositions in pre-FMT and post-FMT fecal samples. “Bef” represents pre-FMT samples; “aft” represents post-FMT samples. **(A)** ANOSIM was used to determine the beta diversities of 18 patients’ gut microbiomes pre- and post-FMT. The bacterial community compositions differed significantly before and after FMT (*p* = 0.017). **(B)** PCoA showed an overall shift in the 18 patients’ gut microbiota compositions after FMT. **(C)** Genus-level microbial compositions of the top 20 abundant taxa in patients’ and donors’ stool samples.

The microbial alpha diversity was increased 4 weeks post-FMT (Figure 1). The Shannon and Simpson indexes were significantly lower in the pre-FMT samples and increased significantly in the post-FMT samples (*p* = 0.0120 and 0.0208, respectively). ANOSIM of the beta diversity revealed that the bacterial community compositions differed significantly between pre-FMT and post-FMT samples (*R* = 0.077, *p* = 0.017; Figure 2A). Principal-coordinate analysis (PCoA) revealed an overall shift in the bacterial community compositions of all patients and donors (Figure 2B). Figure 2C shows the genus-level microbial compositions of the top 20 abundant taxa in both the patient and donor stool samples.

### Bacteria Significantly Changed and Were Correlated With Clinical Symptoms

**FIGURE 3:**
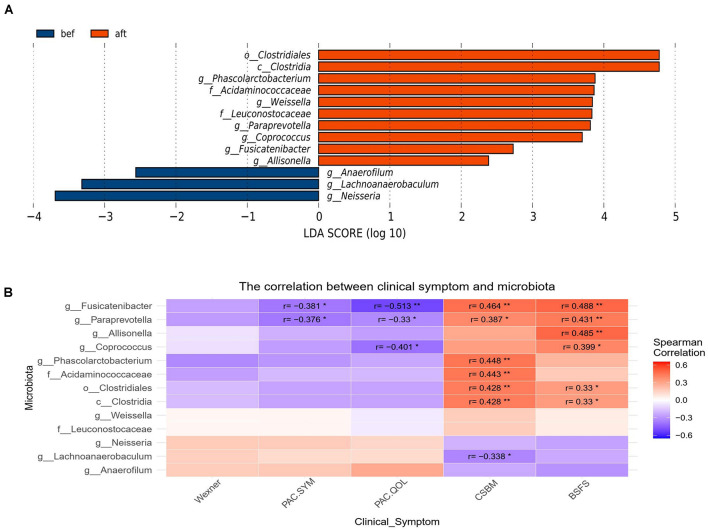
**(A)** LEfSe analysis revealed significant changes in the fecal microbiota compositions before and after FMT. **(B)** Spearman correlation analysis revealed a correlation between patients’ clinical symptoms and the significantly altered bacteria from the LEfSe analysis. *Fusicatenibacter*, *Paraprevotella*, *Allisonella*, *Coprococcus*, *Phascolarctobacterium*, Acidaminococcaceae, Clostridiales, and class Clostridia were correlated with relief of patients’ constipation symptoms, and *Lachnoanaerobaculum* may be correlated with constipation severity (“*” represents 0.01 ≤ *p* < 0.05; “**” represents 0.001 ≤ *p* < 0.01).

LEfSse analysis revealed significant changes in the fecal microbiota compositions. The genera *Paraprevotella*, *Weissella*, *Coprococcus*, *Phascolarctobacterium*, *Allisonella*, and *Fusicatenibacter;* families Acidaminococcaceae and Leuconostocaceae; class Clostridia and order Clostridiales were more abundant in the post-FMT samples. The genera *Lachnoanaerobaculum*, *Anaerofilum*, and *Neisseria* were more abundant in pre-FMT samples (Figure 3A). Spearman correlation analysis revealed that *Fusicatenibacter*, *Paraprevotella*, *Allisonella*, *Coprococcus*, *Phascolarctobacterium*, Acidaminococcaceae, Clostridiales, and Clostridia were correlated with a relief of patients’ constipation symptoms, and *Lachnoanaerobaculum* may be correlated with constipation severity (Figure 3B). Of the above bacteria, *Fusicatenibacter* and *Paraprevotella* were correlated with more than three clinical scales, which might indicate a possible key role of these genera in the efficacy of FMT. We found no significant or coincident relationship between alpha diversity and clinical scores.

### Fecal Micriobiota Transplantation Success Rates Might Be Related to Key Microbiota Abundances

**FIGURE 4:**
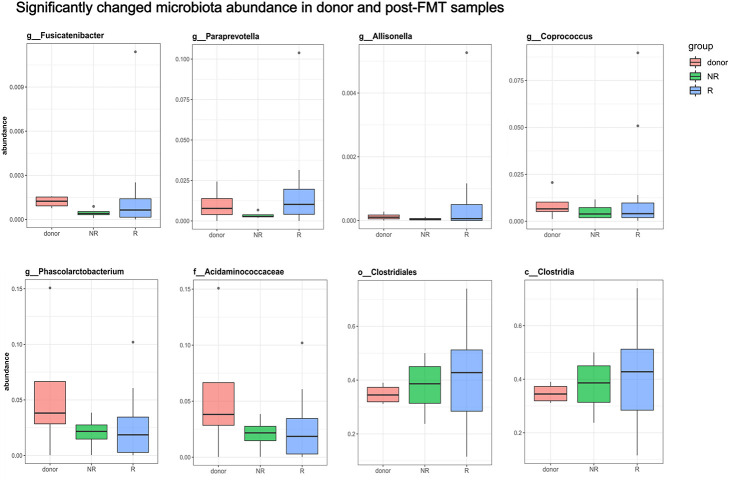
Comparisons of the significantly altered bacterial abundances among donors and post-FMT fecal samples. The compared bacteria were shown to be significantly enriched in post-FMT samples determined by LEFse analysis and meanwhile correlated to clinical symptoms. All 4 donors and 18 patients were included. Group R refers to post-FMT samples from patients who were clinically cured after FMT; Group NR refers to post-FMT samples from patients who were not cured by FMT. Wilcoxon tests showed no significant differences among the groups, but the abundances of *Fusicatenibacter* and *Paraprevotella* were lower in the NR group and higher in the donor and R groups.

**FIGURE 5:**
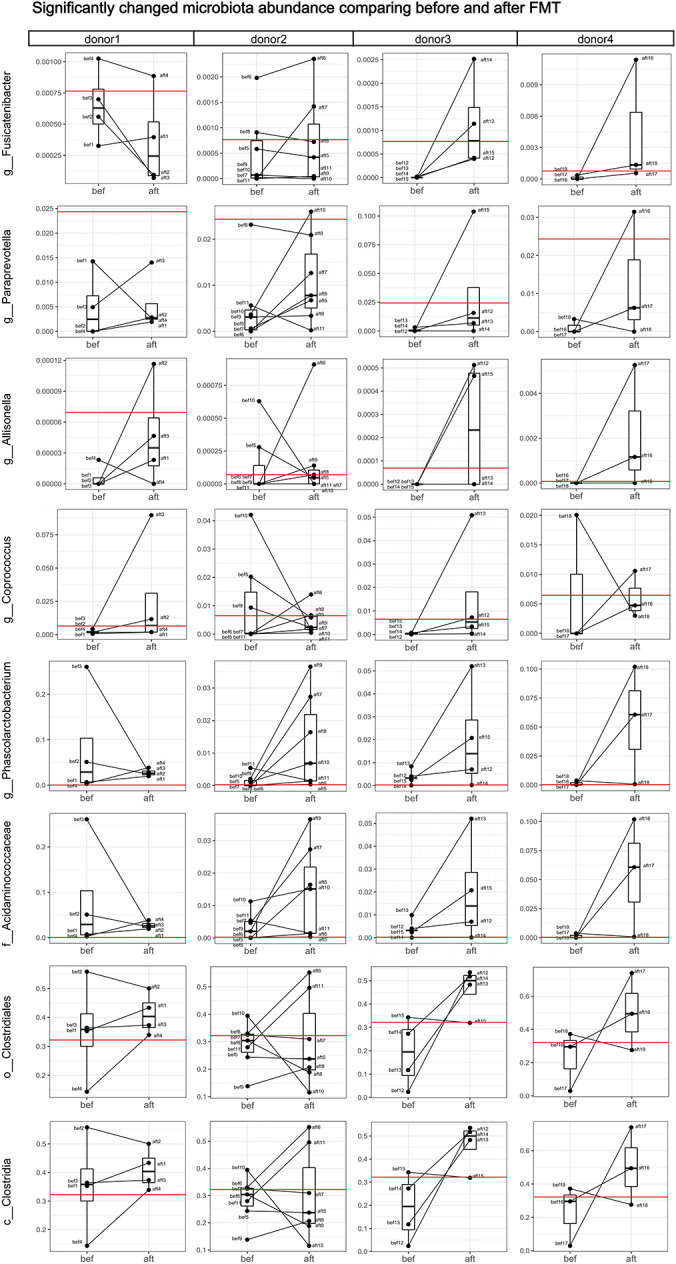
Analyses of abundance changes in post-FMT-enriched bacteria in each donor-recipient pair. Boxplots comparing the bacterial abundances before and after FMT are shown. Each black dot represents one sample (patient); pre-FMT and post-FMT samples from the same patient are connected by a black line. Red horizontal lines represent the donor. One failed patient had a decreased abundance of *Paraprevotella* after FMT, and failed Patients 2, 4, and 5 had decreased abundances of *Fusicatenibacter* after FMT. The recipients of Donors 1 and 2 had more decreased bacterial abundances compared with the increased bacterial abundances in the recipients of Donors 3 and 4.

**FIGURE 6:**
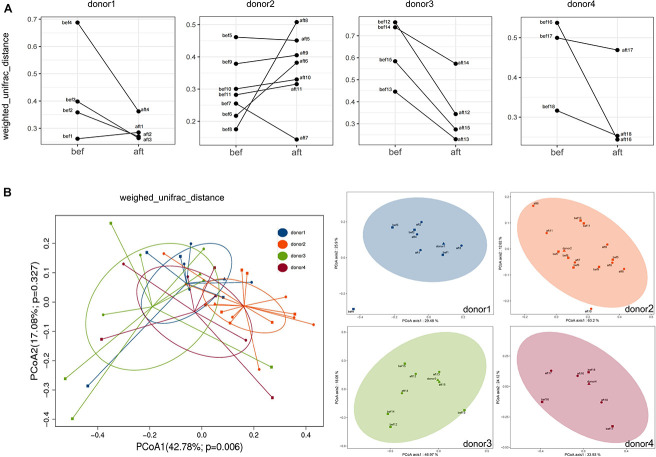
Weighed UniFrac distances of each donor-recipient pair before and after FMT. **(A)** Each graph shows the changes in distance between recipients and their donor. Fecal microbial profiles of most recipients of Donor 1 and all recipients of Donors 3 and 4 became more similar to those of their donor, while most recipients of Donor 2 showed minimal change or became less similar to those of their donor. **(B)** PCoA of the microbiota compositions based on UniFrac distances showed the overall beta diversities of the 4 donors and the pre- and post-FMT samples of the 18 patients (left) and of each donor-recipient group (right).

In this study, the success rates of the recipients differed significantly by donor (*p* = 0.0252). The success rates of the four donors were 25% (1/4), 85.71% (6/7), 100% (4/4), and 100% (3/3). Although the small sample size per donor might contribute to bias in the success rates, we attempted to find a relationship between the donor-recipient gut microbiotas and FMT efficacy. We compared the abundances of the significantly altered bacteria (Figure 3) among the four donor stool samples and the post-FMT samples of the 14 patients whose symptoms were alleviated by the FMT (those who were clinically cured; group R) and the post-FMT samples of the four patients who were not cured by FMT (group NR). Wilcoxon tests showed no significant differences among the three groups; however, the abundances of *Fusicatenibacter* and *Paraprevotella* were lower in the NR group and higher in the donors and R group (Figure 4). We analyzed the abundance alterations of the post-FMT-enriched bacteria in each donor-recipient pair (Figure 5). One NR patient had a decreased abundance of *Paraprevotella* post-FMT, and three NR patients had decreased abundances of *Fusicatenibacter* post-FMT. Compared with the increase trend of those mentioned bacteria abundance in donor 3 and donor 4 recipients, donor 1 and donor 2 seemed to have more decreases. To determine whether the recipients’ fecal microbiota profiles were similar or dissimilar to those of the donors post-FMT, we calculated the weighted UniFrac distances between each donor-recipient pair (Supplementary Table 3). Figure 6 shows the changes in weighted UniFrac distance before and after FMT and the PCoAs of the microbiota compositions based on UniFrac distance. Fecal microbial profiles of most recipients of donor 1 and all recipients of donors 3 and 4 became more like those of the donor, while most recipients of donor 2 changed little or became dissimilar to those of the donor. No definitive regularity existed between the donor-recipient distances and FMT efficacy for FC patients.

### Changes in Short Chain Fatty Acids, Inflammatory Factors, and Correlations With the Fecal Microbiota

**FIGURE 7:**
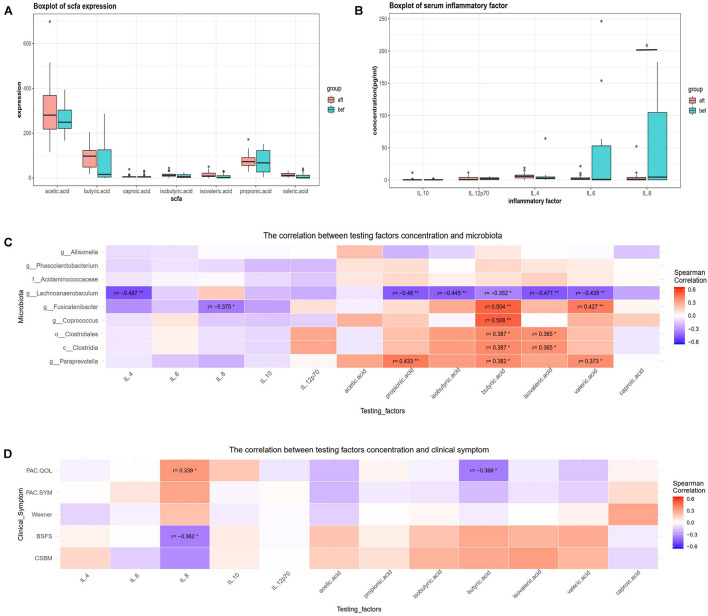
**(A)** Post-FMT and pre-FMT fecal SCFA concentrations of 18 patients were compared using paired-samples Wilcoxon tests. Butyric acid concentrations increased after FMT but not significantly. **(B)** Post-FMT and pre-FMT serum inflammatory factors of 18 patients were compared using paired-samples Wilcoxon tests. IL-8 levels decreased significantly after FMT. **(C,D)** Spearman correlation analysis of pre-FMT and post-FMT clinical symptoms, fecal microbiotas, SCFAs, and serum inflammatory factors of 18 patients revealed that IL-4, IL-8, and some SCFAs (especially butyric acid) were significantly correlated with some bacterial changes. Only IL-8 and butyric acid were correlated with clinical symptoms (“*” represents 0.01 ≤ *p* < 0.05; “**” represents 0.001 ≤ *p* < 0.01).

No significant differences were found in SCFA concentrations between the pre-FMT and post-FMT fecal samples; however, the butyric acid concentrations increased after FMT (Figure 7A). Serum levels of IL-8 were significantly lower after FMT, while no obvious changes were detected in the IL-4, IL-6, IL-10, or IL-12p70 levels (Figure 7B). Correlation analysis showed that IL-4, IL-8, and some SCFAs were correlated with the abundances of some significantly altered bacteria, and the changing trends coincided with severity of patients’ clinical symptoms (Figure 7C). *Paraprevotella* was positively correlated with propionic acid, butyric acid, and valeric acid. *Fusicatenibacter* was positively correlated with butyric acid and valeric acid and negatively correlated with IL-8. Among these SCFA and inflammatory factors, only IL-8 and butyric acid concentrations were significantly correlated with clinical symptoms (Figure 7D).

## Discussion

This retrospective study was conducted to evaluate the clinical efficacy and remodeling of the gut ecology after FMT for constipation. Several articles have reported that FMT can relieve constipation, but few have reported the changes in the gut microbiota post-FMT (Tian et al., 2017; Ding et al., 2018). Using 16S rRNA amplicon sequencing, Ohara (2019) identified 22 microorganismal species that had colonized in recipients 1 month post-FMT; however, whether the colonization was correlated with clinical improvement is unknown. To our knowledge, our study was the first to characterize the correlation between gut microbiota alterations and relief of clinical symptoms after FMT. We thus proposed the specific bacteria and potential mechanism that might have contributed to the clinical efficacy.

Gut dysbiosis exists in patients with constipation and may play an important role in disturbing colonic motility. Simpson indexes have revealed lower alpha diversities for constipated individuals, which is consistent with our findings that alpha diversity increased after FMT (Huang et al., 2018; Tian H. et al., 2020). Some bacteria, including *Parabacteroides* and *Bifidobacterium*, were more abundant in constipated patients’ fecal samples compared with those of healthy individuals. These bacteria have been consistently reported in constipated patients, and other bacteria, such as *Streptococcus* and *Ruminococcus*, have been sporadically reported (Huang et al., 2018; Tian H. et al., 2020). SCFAs, especially butyrate, are also altered in constipated patients, which may affect gut motility and have important immunomodulatory functions (Martin-Gallausiaux et al., 2021). Gut dysbiosis in patients with FC as well as other functional bowel dysmotility disorders, including IBS, may disturb the intestinal immunity, cause “leaky gut,” and affect systemic inflammatory conditions. Some studies reported higher concentrations of proinflammatory cytokines such as IL-6, IL-8, and IL-12 in adult IBS patients. Serum IL-6 and IL-12 levels were also higher in constipated children than in healthy controls. As anti-inflammatory cytokines, IL-10 levels were decreased, and IL-4 levels were increased in IBS patients (Cıralı et al., 2018). In our study, serum IL-8 was decreased in FC patients after FMT, indicating that FMT might have an anti-inflammatory effect that can modulate intestinal motility or may result from recovery of the gut microbiota and motility.

The gut microbiota may cause gut motility disorders via complex mechanisms, and the potential key bacteria differ among studies. Tian Y. et al. (2020) analyzed fecal microbiota changes in constipated patients undergoing FMT and found higher abundances of *Bacteroides* and *Enterobacteriaceae* pre-FMT and increased abundances of *Prevotella* and *Acidaminococcus* post-FMT. Bacteria that were abundant in constipated patients (compared with healthy individuals) or pre-FMT (compared with post-FMT) fecal samples might have a causative effect; however, previous studies did not perform correlation analyses between the abundances of certain microbes and the severity of clinical symptoms. Additionally, some bacteria including *Parabacteroides*, *Bifidobacterium*, *Bacteroides*, and Enterobacteriaceae, were abundant in patients with anxiety and/or depression (Simpson et al., 2021). Because many constipated patients have emotional problems, and these patients were not excluded from the above studies, some of the bacteria may not have played causal roles in colonic motility disturbances during constipation. Further, the bacteria that increased post-FMT may have had no real curative effect on slow colonic motility. In our study, we analyzed the microbiota changes before and after FMT, and examined correlation analyses of certain bacteria, clinical symptom severity scores, and changes in SCFAs and inflammatory factors in constipated patients without symptoms of depression, anxiety, or systemic diseases. Our findings may provide a more compelling hypothesis that certain bacteria, such as *Fusicatenibacter*, *Paraprevotella*, and *Lachnoanaerobaculum*, may help regulate colonic motility, and the mechanism may be related to modulating the fecal butyrate and serum IL-8 concentrations.

FMT is reported to be effective for treating many diseases; however, it is difficult to standardize or improve for disease-specific therapies. We found that colonization of key bacteria, such as *Fusicatenibacter* and *Paraprevotella*, may influence the success of FMT for treating FC, but we found no regularity in donor-recipient distances. Owing to the limitations of a small sample size and short follow-up, other bacteria may be correlated with gut motility regulation; thus, the role of similarity between the donor-recipient pairs requires further research. Functional species screening and mechanistic research are needed to enhance the efficacy of microbial-targeting treatments and make breakthroughs in FMT. Future research should include large datasets, machine learning, and multiomic detection to discover the principles underlying microbiota-host interactions. Mechanistic studies, such as germ-free animal studies, will help verify microbial functions to promote development of improved FMT and precise therapy.

## Conclusion

In summary, we assessed the clinical efficacy and microbial remodeling ability of FMT on constipated patients, together with changes in the fecal SCFAs and systemic inflammatory conditions. FMT relieved constipation symptoms and altered the fecal microbiota compositions to a higher alpha diversity. *Fusicatenibacter*, *Paraprevotella*, *Allisonella*, *Coprococcus*, *Phascolarctobacterium*, Acidaminococcaceae, Clostridiales, and class Clostridia were more abundant in post-FMT fecal samples, which was consistent and in accord with the relief of constipation symptoms. *Lachnoanaerobaculum* was correlated with constipation severity and might play a role in causing constipation. The efficacy of FMT for treating FC might be correlated with the abundances of key bacteria such as *Fusicatenibacter* and *Paraprevotella*, but we found no decisive role of donor-recipient distances. Regulating butyrate production might be one potential mechanism by which the microbiota modulates gut motility. The potential role of systemic IL-8 and its relationship with the gut microbiota also deserve further study. Microbial multiomics studies based on big data analyses are needed to screen causal and functional bacteria. Further research on potential mechanisms may enable precise treatments for constipation and other gut motility disorders.

## Data Availability Statement

The datasets presented in this study can be found in online repositories. The names of the repository/repositories and accession number(s) can be found below: https://www.ncbi.nlm.nih.gov/sra/, PRJNA732583.

## Ethics Statement

The studies involving human participants were reviewed and approved by the Ethics Committee of Shanghai Tenth People’s Hospital of Tongji University. The patients/participants provided their written informed consent to participate in this study.

## Author Contributions

XZ, NL, QC, and HQ conceived, designed the study, and interpreted the results. XZ, NL, and QC collected the samples. XZ and QC performed the laboratory assays and bioinformatics analyses. XZ performed the statistical analysis and drafted the manuscript. NL, QC, and HQ supervised the work and revised and contributed to the final manuscript. NL and HQ contributed with resources and fundings. All authors read and approved the final manuscript.

## Conflict of Interest

The authors declare that the research was conducted in the absence of any commercial or financial relationships that could be construed as a potential conflict of interest.

## Publisher’s Note

All claims expressed in this article are solely those of the authors and do not necessarily represent those of their affiliated organizations, or those of the publisher, the editors and the reviewers. Any product that may be evaluated in this article, or claim that may be made by its manufacturer, is not guaranteed or endorsed by the publisher.

## Footnotes

1
http://rdp.cme.msu.edu/
